# The vocal origin of musical scales: the Interval Spacing model

**DOI:** 10.3389/fpsyg.2023.1261218

**Published:** 2023-10-06

**Authors:** Steven Brown, Elizabeth Phillips

**Affiliations:** Department of Psychology, Neuroscience and Behaviour, McMaster University, Hamilton, ON, Canada

**Keywords:** music, scale, Interval Spacing model, vocal, pitch

## Toward a vocal model of music evolution

The fields of music cognition and psychoacoustics argue that Western musical scales are “natural” because they are derived from the physics of sound via the harmonic series (Rameau, 1722; Helmholtz, 1877; Gill and Purves, 2009). Harmonicity-based theories of music are predicated on the idea that, because common Western scale-intervals are specifiable as simple harmonic ratios (e.g., 3:2 for the perfect fifth), they must be given to us by nature. We can conceive of this graphically as a *linear grid* that is populated along its length by a series of perfect ratios as discrete points (3:2, 4:3, 5:4, etc.), kind of like a number line. Given the fact that this grid is defined a priori by the physics of sound, all that is left for us to do is tune our instruments to these harmonic ratios and …voilà…we have music evolution! In reality, the elusive evolutionary mechanism that allows an acoustic process to generate the corresponding motor capacity to produce scaled pitches is never explained by proponents of the harmonicity theory. The theory is thus confined to the auditory system and its perceptual mechanisms. In addition, the theory is completely asocial, offering no explanation for the evolutionary functions of music in humans, not least for music's universal connection with group performance and the communication of emotion (Brown, 2000, 2022).

An alternative to the accepted view that music is an accommodation to the *perception* of sound is our proposal that music is an accommodation to the *production* of vocally-generated sounds during social communication, as enabled by novel evolutionary changes to the neuro-laryngeal system. While we are unable to state with certainty that the voice was the original musical instrument, we will base our theorizing on the plausible assumption that evolutionary changes to the vocal mechanism led to the emergence of both music and speech. In the case of speech, nobody would argue that surrogates for the voice (such as drums or whistles) evolved first, and yet virtually all theories of musical scales over the last 2,500 years have only ever considered *musical instruments* as the proper model of music's evolution, leading to the emergence of mathematical tuning theories of scales in all of the large civilizations over the last two millennia (Rameau, 1722; Helmholtz, 1877). In such theorizing, scales come first, and melodies are generated to accord with them, just as with modern-day symphony orchestras. In contrast, a vocal-motor account argues that melodic vocalizations evolved long before cultural evolution of precisely tunable musical instruments permitted theoretical formulations of scales.

**Figure 1:**
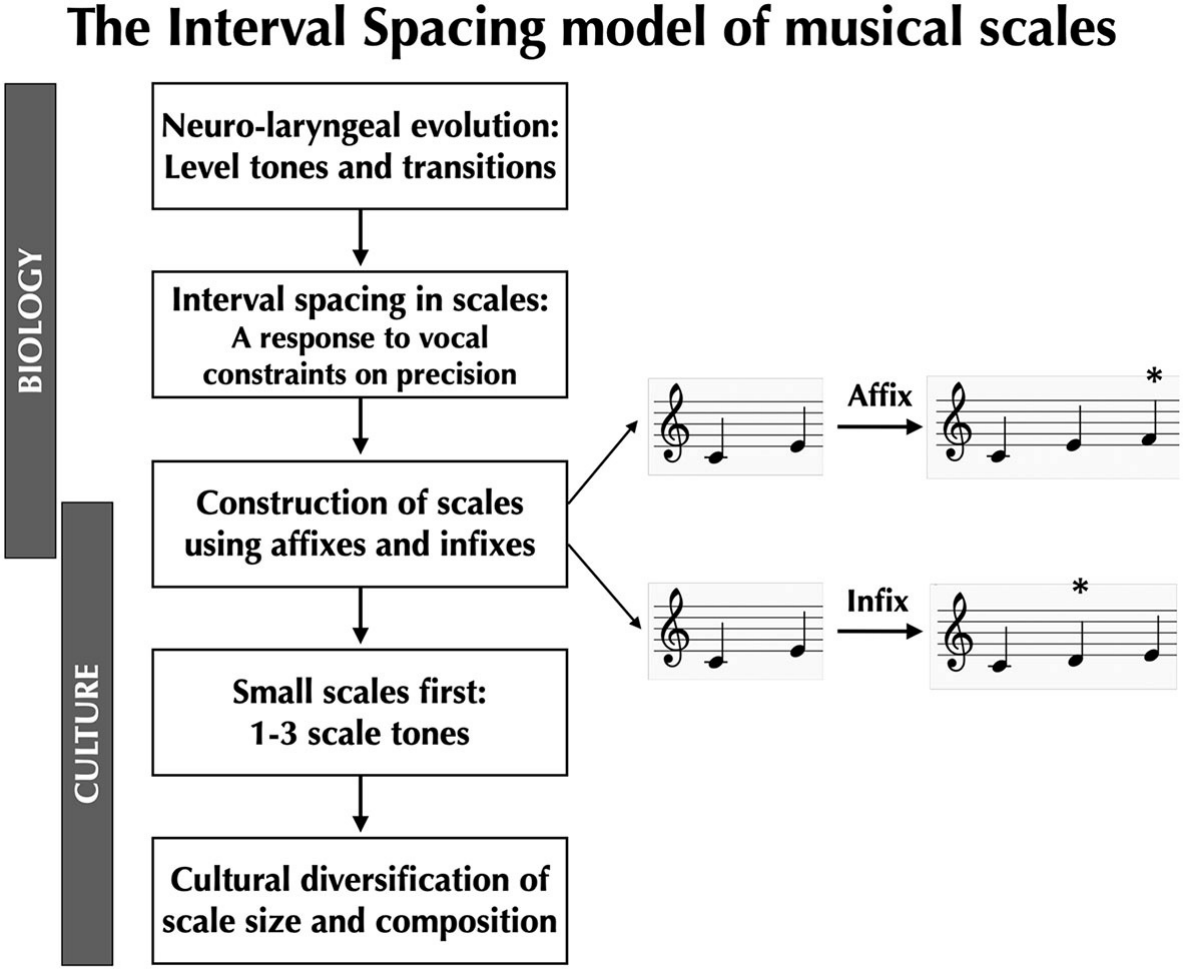
The Interval Spacing model of musical scales. Coupled evolutionary changes to the larynx and the larynx-controlling region of the primary motor cortex confer onto humans the capacity to produce level tones (“pitches”) and specific intervallic transitions between them, allowing humans to sing musical melodies. However, the voice is a highly imprecise pitch-generating instrument, necessitating that there be adequate spacing between adjacent scale-tones in order for these tones to be distinguishable in production. Next, Sachs (1943) proposed that scales can be constructed from the bottom up through a process of accretion. He proposed two mechanisms for achieving this: (1) affixation, where pitches are added to one end of an existing scale, and (2) infixation, where pitches are added internal to an existing scale (the ^*^ indicates the newly added scale tone). Sachs also proposed that small scales were the first scales to appear during human evolution, and that larger scales appeared later. Cultural diversification of scale-types through changes in scale size and composition can occur in a generative manner using a small set of building blocks, most notably semitones, whole tones, and minor thirds.

Instead of basing a theory of musical scales on a prescribed top-down grid of harmonic ratios, we need to start with the bottom-up mechanisms of vocal production, not least since the voice cannot be tuned a priori. These mechanisms are evolutionarily novel in the human lineage, and so they provide critical insights into why human music is such a distinct phenomenon in nature, and why similar melodic systems based on scaled pitches are so uncommon in other animals, despite similarities in their auditory organs. In the following sections, we will present a vocal model of the evolutionary origin of musical scales called the Interval Spacing model (Pfordresher and Brown, 2017; Brown, 2022; Phillips and Brown, 2022a,b; see Sato et al., 2019 for a related idea). This is summarized in Figure 1. According to this model, what is “natural” in music is that which is given to us by the biology of vocal production, not by the physics of sound perception, although the two processes unquestionably condition one another, just as in all sensorimotor systems (Prum, 2012, 2013).

## Neuro-laryngeal evolution

A vocal model of the origin of music creates a linkage to the significant biological changes that have occurred to both the vocal tract and the vocal regions of the cerebral cortex during the course of human evolution. Not only is the larynx in a descended position in the vocal tract compared to all non-human primates (Fitch, 2000; Nishimura, 2006), but the cortical region that controls vocal pitch through its regulation of the laryngeal muscles occupies a novel location in the human motor cortex compared to the monkey brain (Brown et al., 2008). In fact, humans have evolved a *dual representation* of the larynx in the motor cortex. One area is most likely the homolog of the monkey area, whereas the other is an evolutionarily novel human area for the control of vocal pitch in speech and song (Brown et al., 2008; Pfenning et al., 2014; Belyk and Brown, 2017). This latter region–called the larynx motor cortex (LMC)–most likely conferred onto humans the capacity for voluntary control of vocalization, compared to the far more involuntary systems of non-human primates. These linked evolutionary changes to the larynx and the vocal brain have contributed to the signature feature of human vocalization relevant to music, namely the ability to produce level tones (“pitches”) coupled with the ability to produce stable intervallic transitions between these pitch-levels to generate melodies. Without such a mechanism, one could not talk about pitch classes, interval classes, scales, or melodies in music. Music in most cases is a digitization of a continuous acoustic space to create stable–if imprecise–pitch-levels and the intervals between them.

The other significant evolutionary change to the human vocal system beyond the ability to phonate level tones and transitions is our capacity for vocal learning, another offshoot of the evolution of voluntary control of vocalization. Vocal production learning is a uniquely human trait among all living primates and is rare among animals more generally (Petkov and Jarvis, 2012; Vernes et al., 2021). Importantly, this newly evolved capacity for vocal imitation has an impact not only on how individuals learn how to produce music, but on how groups of people chorus together. Chorusing is a social feature of music that is rarely mentioned in models of music evolution, although Jordania (2006) provides a comprehensive counterexample. It is an important phenomenon to consider in music's evolution since it provides the appropriate context to think about harmonicity's role in the origins of music, namely *harmonizing*. We need octave equivalence in order to understand how people of differing vocal ranges are able to sing in unison, despite singing pitches that are an octave apart (Sato et al., 2019). Octave equivalence might in fact be harmonicity's most salient contribution to music.

## Interval spacing as a response to vocal imprecision

While music is defined by the use of relatively discrete pitch-levels and transitions, a vocal model of the origins of music has to contend with the great deal of *imprecision* that characterizes the voice as a pitch-producing instrument, not least compared with the tunable instruments that serve as the foundation for harmonicity theories. While the 3:2 ratio of the perfect fifth and the 4:3 ratio of the perfect fourth are discrete points along the linear grid of harmonic ratios, the production of these interval-classes during singing tends to show overlap. Pfordresher and Brown (2017) used the metaphor of an “interval island” to describe interval-classes during singing and how these islands overlap one another in actual vocal production.

Phillips and Brown's (2022b) computational analysis of 418 scales from indigenous vocal traditions across 10 world regions demonstrated a mean imprecision value of 1.5 semitones across all pitch-classes. Such a level of imprecision implies that scale spacings of less than a whole tone (i.e., 2 semitones) are going to be overlapping. This 1.5-semitone imprecision leads to an upper limit on the number of scale tones that can reasonably fit within an octave, resulting in heptatonic scales as the limiting case. This raises the important point that a vocal model of music is able to account for the *size* of scales cross-culturally. The harmonicity theory of scales, being based on perception alone, is only limited by the 4-cent just-noticeable difference observed in frequency perception (Oxenham, 2013), hence creating the possibility for scales to have 300 pitches per octave (where an octave is 1,200 cents). A motor theory based on vocal imprecision explains why such a model is untenable. A follow-up analysis by Brown et al. (in preparation) of the same set of 418 scales analyzed by Phillips and Brown (2022b) revealed that the mean interval-size between adjacent scale-tones was 2.2 semitones, or just larger than a whole tone. In fact, more than 90% of the scale-intervals in the corpus spanned the region of 50 to 350 cents, corresponding with three step-sizes in Western music theory: a semitone, whole tone, and minor third, in other words a whole tone +/- a semitone. The remaining 10% of the scale-intervals were made up of the major third and perfect fourth.

We have capitalized on such findings to create a model of musical scales called the Interval Spacing model, as summarized in Figure 1 (Pfordresher and Brown, 2017; Phillips and Brown, 2022a,b). The central tenet of the model is that physiological constraints on vocal production have had a causal impact on the size and intervallic spacing of musical scales. Adjacent scale-tones have to be spaced far enough apart to be distinguishable in production, but not so far as to tax the vocal system. As a result, the most common scale interval cross-culturally is the whole tone (Mehr et al., 2019; Brown et al., in preparation). The semitone seems to be the smallest reliably singable interval in music (Burns, 1999). Cultural factors in singing style–including the use of portamento, vibrato, and melisma–can further push vocalists away from precise intervallic production (Lomax, 1968; Wood, 2021), even while melodic traditions tether their songs to certain structural regularities, creating fuzzy “zone scales” (Kondrat'eva, 2009) or “loosely-knit modal folk-song scale[s]” (Grainger, 1908).

## Construction of scales from the bottom up

Can we imagine an alternative mechanism for generating musical scales other than the top-down grid of harmonic ratios given to us by acoustics? Can we imagine a model of scales that prioritizes melodies over scales and that sees scales as an abstraction of the way that people sing melodies (Meyer, 1956)? While the Interval Spacing model accounts for the impact of vocal constraints on the composition of scales (i.e., whole tones are optimal) and the size of scales (i.e., heptatonic or smaller), it does not provide an evolutionary explanation for how scales are constructed or how they diversify culturally. To shed light on this topic, we revive a bottom-up scale model put forward by the comparative musicologist Curt Sachs in the 1940′s. This is shown in the middle panel of Figure 1. According to Sachs (1943), scales evolve by the *accretion* of scale tones. Scales start out small and increase in size over historical time up to their physiological vocal limit of around seven pitches per octave. Sachs proposed two mechanisms by which scales can expand in size: affixation and infixation (see Figure 1). In affixation, a new pitch is added to one end or the other of an existing scale. In infixation, a new pitch is added internal to an existing scale as a filler. Note that the Interval Spacing model provides guidelines for how these new pitches should be incorporated into the existing scale. Whether a scale expands through affixation or infixation, the spacing between adjacent scale-tones should generally be a whole tone +/- a semitone, as is clearly seen in indigenous vocal scales cross-culturally (Brown et al., in preparation).

Sachs' model is a generative theory of scales: it establishes a combinatorial mechanism for the formation of scales. Scales can expand using intervals such as semitones, whole tones, and minor thirds as their basic building blocks. This can create what Sachs (1943) calls “a kaleidoscopic infinity of variations and permutations” (p. 39), as shown empirically by the striking amount of scale diversity both within and between cultures. although cultural evolution can stabilize certain patterns, for example the pentatonic scale (Savage et al., 2015). The Indian system of Karnatic *melakarta* scales is similarly a combinatorial system based on this same set of building blocks (Massey and Massey, 1993). Note that this generative model of scale construction contrasts with the harmonicity model in that the latter views the octave as its starting point and then takes a *divisive* approach to breaking down the octave through the sequential incorporation of discrete harmonic ratios (e.g., 3:2, 4:3). By contrast, the Sachs system is an *additive* mechanism based on the accretion of scale tones (and potentially the subtractive loss of scale tones as well). It assumes neither an octave nor a tonic, but simply the sequential addition of stably spaced tones. In addition, Sachs (1943) proposed that small scales evolved before larger scales during the course of human evolution, and this seems to be a reasonable working hypothesis. In Brown et al. (in preparation), 12% of the scales in the corpus were three tones or less.

An additive model might suggest that musical scales should be equidistant, as based on some optimal interval size. For example, McBride and Tlusty (2020) provide a computational analysis that argues that a model like the Interval Spacing model predicts equidistance in scales. Interestingly, Sachs (1943) does not broach this topic. While most of the transcriptions that he presents of scales from indigenous songs are non-equidistant (e.g., CFG, CFA), some of them are indeed equidistant (FGA, DGC). Musicological work has demonstrated the presence of equidistant scales in both vocal (Ambrazevičius, 2009; Ambrazevičius and Budrys, 2013) and instrumental music (Ross and Knight, 2019; McBride and Tlusty, 2020). A commonly described limitation of equidistant scales is the presumed absence of a tonal center. However, Ross and Knight (2019) describe mechanisms by which this limitation can be successfully overcome.

In addition to the experimental (Pfordresher and Brown, 2017) and ethnographic (Phillips and Brown, 2022b) approaches discussed here, future work on the Interval Spacing model should examine topics such as the modeling of scales based on interval-spacing principles (e.g., avoid consecutive semitones), the analysis of pitch-intervals in spoken utterances (see Chow and Brown, 2018), the ability of people to sing melodies that either abide by or violate interval-spacing principles, and ontogenetic analysis of how musical scales emerge developmentally in children's singing, where children are well-known to be imprecise singers (Goetze et al., 1990; Rutkowski, 1990; Welch, 2009).

In conclusion, the Interval Spacing model's basic tenet that vocal-motor constraints are a major causal factor in explaining the known scale-tone spacings of “a whole tone +/- a semitone” coupled with Sachs' generative model for constructing scale sequences from the bottom up provide a production-driven and vocal alternative to the standard perception-driven theory of scales based on perfect harmonic ratios and the a priori tuning of instruments. These ideas are supported by the evidence of striking evolutionary changes to the neuro-laryngeal system in humans, establishing music, like speech, as a novel biological function.

## Author contributions

SB: Conceptualization, Funding acquisition, Writing–original draft, Writing–review and editing. EP: Writing–review and editing.
